# Clinical characteristics of H‐type hypertension and its relationship with the *MTHFR* C677T polymorphism in a Zhuang population from Guangxi, China

**DOI:** 10.1002/jcla.23499

**Published:** 2020-08-13

**Authors:** Liu Qiang Huang, Chong Xin Wu, Hua Qing Wei, Ge Xu

**Affiliations:** ^1^ Department of Cardiology The Affiliated Wuming Hospital of Guangxi Medical University Nanning China; ^2^ Department of Cardiology The first Affiliated Hospital of Guangxi Medical University Nanning China

**Keywords:** hyperhomocysteinemia, hypertension, MTHFR, polymorphism, Zhuang nationality population

## Abstract

**Objective:**

This study was designed to assess the clinical presentation of patients with H‐type hypertension who were of Zhuang nationality in Guangxi, China. The relationship between the C677T polymorphism in the MTHFR gene and H‐type hypertension was also assessed.

**Methods:**

This was a case‐control study in which 185 Zhuang nationality patients with hypertension that had been hospitalized at the Wuming Hospital of Guangxi Medical University between February 2018 and December 2018 were assessed for plasma homocysteine (Hcy) levels. These levels were used to divide patients into H‐type (>15 μmol/L) and non‐H‐type (≤15 μmol/L) hypertension groups. Patient clinical data were then analyzed, and PCR was used to analyze samples from all patients for the presence of the C677T polymorphism in the MTHFR gene. Differences between these two groups of hypertension patients were then compared using appropriate statistical methods.

**Results:**

We found that relative to patients in the non‐H‐type hypertension group, patients in the H‐type hypertension group exhibited significant differences in sex, age, urea nitrogen levels, creatinine levels, and uric acid levels. There were, however, no significant differences between these two groups with respect to interventricular septum thickness, left ventricular posterior wall thickness, or ejection fraction. We did not detect any association between the MTHFR gene C677T polymorphism and H‐type hypertension in Zhuang nationality individuals in Guangxi.

**Conclusion:**

Risk of H‐type hypertension is not associated with the MTHFR C677T polymorphism in hypertensive individuals of Guangxi Zhuang nationality in China.

## 1 INTRODUCTION

H‐type hypertension is a form of primary hypertension that also presents with hyperhomocysteinemia (HHcy). This condition is relatively common in mainland China and is associated with an elevated risk of stroke.^1^ On average, adult Chinese patients with hypertension have plasma total homocysteine (tHcy) levels of ~15 mmol/L owing to environmental and genetic factors, with elevated plasma Hcy levels being evident in 75% of Chinese hypertension patients, including 91% of male and 63% of female patients.^2^

In Chinese patients with hypertension, elevated Hcy levels are an independent risk factor for stroke.^3^ According to previous studies, for every 5 mmol/L increase in tHcy, the chance of stroke increases by 59%. In addition, there appears to be a synergistic effect between HHcy and hypertension, both of which are associated with an increased risk of cardiovascular disease and stroke.^4^

Elevated tHcy levels are usually caused by vitamin B deficiency, genetic variations, and a significant decrease in renal function. Of known tHcy genetic determinants, the C677T polymorphism of the methylenetetrahydrofolate reductase (MTHFR) gene is by far the most important and best‐studied.^5^, ^6^, ^7^

Methylenetetrahydrofolate reductase is a key regulator of Hcy levels, and the C677T mutation in this gene results in a sharp decline in Hcy activity.^8^ The MTHFR C677T (rs1801133) mutation is relatively common and has been linked to increased blood pressure and hypertension in individuals carrying this mutation.^9^, ^10^

The Zhuang people are the largest minority group in China, and yet there have been few studies conducted to date regarding the characteristics of hypertension in Zhuang populations. Whether MTHFR gene polymorphisms are related to hypertension in Zhuang populations thus remains unknown. Herein, we therefore examined the relationship between clinical findings, cardiac ultrasound results, and plasma Hcy levels in 185 patients of Zhuang nationality suffering from hypertension that had been hospitalized between February 2018 and December 2018 at the Wuming Hospital affiliated with Guangxi Medical University. These patients additionally underwent genotyping to determine whether they were carriers of the C677T polymorphism, and the relationship between this polymorphism and the abovementioned variables was also assessed.

## 2 MATERIALS AND METHODS

### 2.1 Study population

In total, 185 patients of Zhuang nationality with hypertension that presented to Wuming Hospital affiliated with Guangxi Medical University between February 2018 and December 2018 were enrolled in the present study. Patients were an average of 66 years old, with 108 being male and 77 being female (58.38% and 41.62%, respectively). Patients were enrolled if they met the following criteria: (a) diastolic blood pressure (DBP) ≥ 90 mm Hg or systolic blood pressure (SBP) ≥ 140 mm Hg; (b) Patients were of Zhuang nationality and presented at the study hospital. Patients suffering from the following conditions were excluded from enrollment in this study: chronic CVD, secondary hypertension, hypercalcemia, chronic cerebrovascular disease, pregnancy, and chronic liver or kidney disease. The Ethics Committee of Guangxi Medical University approved the study, and all participants provided consent to participate after being informed of the study protocol.

### 2.2 Physical examination and laboratory analyses

Patient baseline demographic and clinical characteristics including sex, age, weight, and whether or not patients had been diagnosed with coronary heart disease, diabetes, or cerebral infarction were recorded. In order to measure blood pressure, patients were instructed to rest while for 5 minutes, after which a sphygmomanometer was used to determine blood pressure in the right arm. For blood collections, patients fasted overnight for a minimum of 12 hours, after which blood was collected for measurements of high‐density lipoprotein cholesterol (HDL‐C), low‐density lipoprotein cholesterol (LDL‐C), creatinine, urea nitrogen, and uric acid. HDL‐C and LDL‐C were measured using an AU5800 autoanalyzer (Beckman Coulter). Creatinine, urea nitrogen, and uric acid were detected with an automatic analyzer (Hitachi 7080, Hitachi). In addition, Hcy levels were measured via enzyme‐linked immunosorbent assay (ELISA). All tests are carried out in accordance with provided instructions.

### 2.3 MTHFR C677T genotyping

In order to determine whether patients were carriers of the MTHFR C677T polymorphism, whole blood samples collected using EDTA as an anticoagulant were used for genotyping via Taqman probe technology. In the HapMap database, MTHFR is located from positions chr1:11768374 to 11788702, with C677T being located at position rs1801133. ABI (USA) synthesized the Taqman probe that was used for this genotyping experiment, and automated PCR reactions were conducted using a Roche Applied Science LightCycler 480 instrument.

### 2.4 Statistical analysis

Hyperhomocysteinemia (HHcy) was defined as plasma Hcy >15 mmol/L. Continuous variables are means ± standard deviation. As HOMA‐IR does not correspond to a normal distribution, these values are given as medians and quartiles. Categorical variables are expressed as percentages. Continuous and categorical variables were compared via ANOVAs and chi‐squared tests, respectively. The relationship between MTHFR C677T genotype and plasma Hcy levels in patients was analyzed via multivariate linear regression analysis. A two‐sided *P* ≤ .05 was the significance threshold for these analyses. SPSS (v22.0; IBM Inc) was used for all statistical testing.

## 3 RESULTS

### 3.1 Patient baseline characteristics

In total, 185 patients of Zhuang nationality with hypertension were enrolled in this study and were divided into two groups based upon whether or not their plasma Hcy level was >15 μmol/L, with patients over this threshold being classified as having H‐type hypertension and those below it being classified as having non‐H‐type hypertension. The baseline characteristics of patients in both groups are compiled in Table 1. Patient body weight, SBP, DBP, LDL‐C, HDL‐C, coronary heart disease incidence, diabetes incidence, and cerebral infarction incidence did not differ significantly between groups (*P* > .05). Relative to the non‐H‐type group, the frequency of male patients, average age, and average urea nitrogen, creatinine, and uric acid levels were significantly higher among patients with H‐type hypertension (*P* ≤ .05, Table 1).

**Table 1:** Comparison of general clinical baseline data of study subjects $\left[\bar{x}\pm {\text{s, n, M (Q}}_{R})\right]$

Clinical indicators	Total	H‐type hypertension	Non‐H‐type hypertension	*P* value
Man (n)	185	76	109	–
Sex (n/%)	108 [58.38]	58 [76.32]	50 [45.87]	.00
Age (y)	66 (54‐73)	69.0 (60.25‐76.75)	64 (54‐70)	.01
Weight (kg)	61.44 ± 11.20	61.84 ± 11.97	61.17 ± 10.67	.69
SBP (mm Hg)	159 (138.50‐175.0)	155.5 (138.0‐172.5)	160 (142‐179)	.41
DBP (mm Hg)	86 (72.5‐100.50)	87.5 (73.25‐99.0)	85 (72‐101.5)	.97
LDL‐C (mmol/L)	2.73 (1.90‐3.42)	2.62 (1.75‐3.57)	2.83 (2.01‐3.33)	.70
HDL‐C (mmol/L)	1.26 (1.03‐1.76)	1.24 (1.02‐1.87)	1.28 (1.03‐1.70)	.82
UA (μmol/L)	5.11 (3.87‐6.38)	5.48 (4.43‐6.80)	4.9 (3.67‐6.28)	.01
Creatinine (μmol/L)	94.2 (76.50‐138.10)	109 (88.23‐318.7)	90.3 (67.95‐119.65)	.00
Uric acid (μmol/L)	351.20 (261.40‐466.95)	391.85 (248.05‐491.63)	345.5 (261.4‐424.35)	.05
Coronary heart disease (n)	64 [34.59]	32 [42.11]	32 [29.36]	.07
Diabetes (n)	22 [11.89]	9 [11.84]	13 [11.93]	.99
Brain infarction (n)	40 [21.62]	20 [26.32]	20 [18.35]	.20

### 3.2 Comparison of cardiac ultrasound findings between patient groups

When echocardiographic findings were compared between these two patient groups, we found that there were no significant differences in any of the three measured indicators between patients with H‐type and non‐H‐type hypertension (*P* > .05; Table 2).

**Table 2:** Comparison of cardiac ultrasonography between patients with H‐type hypertension and non‐H‐type hypertension

Clinical indicators	Number (n)	Septal thickness (mm)	Left ventricular posterior wall thickness (mm)	Ejection fraction (%)
Total	185	10 (9‐11)	10 (9‐11)	63 (58‐68)
H‐type hypertension	76	10 (9‐11)	10 (9‐11)	63 (54.25‐67.0)
Non‐H‐type hypertension	109	10 (9‐11)	10 (9‐10)	64 (59‐69)
*P* value		.501	.224	.092

### 3.3 The relationship between the MTHFR C677T polymorphism and Hcy levels

Lastly, we analyzed the relationship between MTHFR C677T allele frequencies and Hcy levels in this hypertensive patient population (Table 3). In patients with H‐type hypertension, MTHFR C677T genotype frequencies for the CC, CT, and TT genotypes were 67.11%, 25%, and 7.89%, respectively, while they were 66.06%, 29.36%, and 4.59% among patients with non‐H‐type hypertension, respectively, with no significant difference between these groups (*χ* ^2^ = 1.14, *P* = .57). Allele frequencies in the H‐type group (C, 40.74%; T, 42.47%) and non‐H‐type group (C, 59.26%; T, 57.53%) did not differ significantly (*χ* ^2^ = 0.072, *P* = .79). These findings thus indicated that the MTHFR C677T polymorphism was not significantly related to elevated Hcy levels in this Guangxi Zhuang nationality population of hypertensive patients (both *P* > .05).

**Table 3:** MTHFR C677T distribution in H‐type hypertension and non‐H‐type hypertension groups

Groups	Genotype frequencies (%)			Allele frequencies (%)	
	CC	CT	TT	C	T
Total (n = 185)	123 (66.49)	51 (27.57)	11 (5.95)	297 (80.27)	73 (19.73)
H‐type hypertension (n = 76)	51 (67.11)	19 (25)	6 (7.89)	121 (40.74)	31 (42.47)
Non‐H‐type hypertension (n = 109)	72 (66.06)	32 (29.36)	5 (4.59)	176 (59.26)	42 (57.53)
	*χ* ^2^ = 1.14, *P* = .57			*χ* ^2^ = 0.072, *P* = .79	

## 4 DISCUSSION

Herein, we found that elevated Hcy levels were associated with increased urea nitrogen, creatinine, and uric acid levels in hypertensive patients of Zhuang nationality in Guangxi, China. However, we did not discover any significant relationship between the presence or absence of the MTHFR C677T polymorphism in this patient population and the risk of having elevated Hcy levels.

The Guangxi Zhuang Nationality Autonomous Region is located in the southern part of China. This region has a higher concentration of ethnic minorities relative to China as a whole. It is home to 11 ethnic minority populations including persons of the Zhuang, Yao, Miao, Maonan, Hui, Jing, and Shui nationalities. Of these populations, the Zhuang nationality is the largest minority ethnic group in China. However, relatively few studies regarding the characteristics of hypertension in persons of Zhuang nationality have been conducted to date. As such, it remains unclear as to whether there are differences between H‐type and non‐H‐type hypertension outcomes in Zhuang nationality patients.

In this study, we assessed the underlying clinical characteristics of a Zhuang study population and analyzed the relevance of the C677T polymorphism as a predictor of HHcy levels (Hcy > 15 mmol/L). Relative to patients with non‐H‐type hypertension, the frequency of male patients (*P* = .00), as well as age (*P* = .01), urea nitrogen (*P* = .01), creatinine (*P* = .00), and uric acid (*P* = .05) levels were significantly higher among patients with H‐type hypertension. However, in this Zhuang population, we found that the C677T polymorphism has poor sensitivity and moderate specificity as a predictor of plasma Hcy levels, indicating that it is not an effective tool that can be used to screen for Guangxi Zhuang patients with H‐type hypertension. The results of this study are somewhat inconsistent with previous results. A meta‐analysis of 114 studies found that C677T polymorphism was associated with hypertension (OR = 1.36, 95% CI = 1.20‐1.53).^11^ In addition, a separate study based on a Chinese population also found that the MTHFR C677T gene polymorphism was associated with a higher risk of H‐type hypertension.^12^ The different study populations in these analyses may explain the observed result inconsistencies, as we herein focused on a Zhuang population, whereas these prior studies have focused on populations of Han ethnicity. This thus suggests that these different ethnic populations may exhibit distinct patterns of genetic susceptibility to H‐type hypertension.

Hyperhomocysteinemia is an independent risk factor associated with both cardiovascular disease and stroke incidence.^13^, ^14^, ^15^ This condition results from elevated tHcy plasma levels and can impact arterial structure and function, thereby increasing the risk of cardiovascular disease, stroke, pre‐eclampsia, and neurological disease.^16^, ^17^, ^18^

Many studies have defined a clear link between HHcy and hypertension, and the underlying mechanism is thought to be linked to the disrupted function of vascular endothelial and smooth muscle cells.^19^, ^20^, ^21^, ^22^, ^23^ Indeed, HHcy can adversely impact endothelial cell function via inducing oxidative damage^24^, ^25^ and can regulate vascular smooth muscle cell proliferation^26^ and accelerate the development of atherosclerosis.^27^ In hypertensive patients, these adverse effects are associated with H‐type hypertension.^28^

The methyltransfer pathway is responsible for Hcy formation from methionine (Met).^29^ In healthy individuals, Hcy levels are maintained in a homeostatic range (5‐15 μmol/L). The MTHFR, MTR, and MTRR proteins are key regulators of a range of important biological pathways including Hcy metabolism. The MTHFR C677T and A1298C polymorphisms can significantly alter the levels of a number of physiologically important metabolites in vivo, including Hcy, folic acid, vitamin B6, and vitamin B12. These metabolites are in turn linked to risk hypertension, stroke, and CVD.^30^, ^31^, ^32^ However, in our study, we detected no significant relationship between the C677T genotype and elevated tHcy levels, suggesting that this mutation is unrelated to an increased risk of HHcy. This result may be a consequence of the genetics, environment, and life history of the study population.

In this study, our sample size was relatively small, and as such we may not have had sufficient statistical power to detect certain associations between variables. Future large‐scale, high‐quality research studies should therefore be conducted in order to validate these findings. It is also important to note that folate deficiency is very common in Chinese populations. We did not measure the levels of plasma B vitamins such as folic acid, nor did we evaluate dietary/supplemental vitamin B intake, thus preventing us from exploring the impact of B vitamin levels on patient phenotypes. Such data may have led to different conclusions regarding the relationship between MTHFR C677T and HHcy. Another limitation of this study is that all studied patients had hypertension, and thus further comparisons and validation of these results in non‐hypertensive populations are still required. Finally, the study was limited to a Guangxi Zhuang population, and the frequency and impact of this MTHFR C677T polymorphism can differ as a function of both ethnicity and location. As such, the relationship between the MTHFR C677T polymorphism and susceptibility to H‐type hypertension needs to be verified in other Chinese populations.

In summary, elevated Hcy levels in patients of Zhuang nationality are associated with elevated urea nitrogen, creatinine, and uric acid levels. These levels are not, however, related to patient MTHFR C677T genotype, indicating that the presence or absence of this polymorphism has no clear bearing on the diagnosis or treatment of hypertension in this ethnic population, nor does it have any known relationship with patient prognosis.
